# Amalgam versus composite restorations: a cost-consequence analysis

**DOI:** 10.1038/s41415-026-9797-1

**Published:** 2026-07-24

**Authors:** Oliver Bailey, Laura Ternent, Simon J. Stone, Christopher R. Vernazza

**Affiliations:** 801263779949917492828 School of Dental Sciences, Newcastle University, Newcastle upon Tyne, United Kingdom; 507071959828993531655 Population Health Sciences Institute, Newcastle University, Newcastle upon Tyne, United Kingdom

## Abstract

**Aims** Amalgam is being phased out globally by 2034. Under English NHS provision, amalgam is predominantly used to restore posterior teeth. A broad evaluation of the comparative costs and consequences of using amalgam versus various composite formulations to restore posterior permanent teeth in adults was therefore required to inform a phase-out.

**Methods** Multiple research strands (a large clinician survey, a discrete choice experiment to obtain public values for relevant aspects of direct restorations, and a model-based cost-effectiveness analysis) alongside new data were synthesised. Costs and consequences to patients, funders and clinicians of using amalgam and direct alternatives over varying timeframes under English NHS provision were quantified in a cost-consequence analysis.

**Results** Differences in tooth survival (4 years; 12 years non-discounted), patient valuation (£33), and lifetime cost to patients (£70 direct; £15 indirect), funders (£34) and clinicians (£83) favour amalgam over composite restorations in English NHS care.

**Conclusions** Except for appearance, all other outcomes and costs favoured the use of amalgam over composites for the direct restoration of posterior teeth for all stakeholders. If amalgam is phased-out in the near future without fundamental change, there are significant implications for the most at need in society and the survival of NHS dental services.

**Supplementary Information:**

Zusatzmaterial online: Zu diesem Beitrag sind unter 10.1038/s41415-026-9797-1 für autorisierte Leser zusätzliche Dateien abrufbar.

## Key points

- Amalgam is still the most used material to restore posterior teeth under NHS provision despite a 2034 phase-out date which justifies this economic evaluation.
- Restoration and tooth survival, patient valuation and lifetime cost data to patients, funders and clinicians all favour amalgam over composite.
- Without fundamental change, there are significant implications for the most at need and the survival of NHS dental services.

**Supplementary Information:**

Zusatzmaterial online: Zu diesem Beitrag sind unter 10.1038/s41415-026-9797-1 für autorisierte Leser zusätzliche Dateien abrufbar.

## Introduction

Dental amalgam use has been phased out in the European Union (EU),^1^ with a global commitment to phase it out by 2034.^2^ Amalgam is still the most used material to restore posterior teeth under National Health Service (NHS) provision in the United Kingdom (UK),^3^^,^^4^^,^^5^ which is likely the main reason the UK did not follow the EU's accelerated phase-out.

Expert reviews have described resin-based composites as the only reasonable alternative in the required time frame.^6^^,^^7^ ‘Composite' encompasses a broad range of materials with varying characteristics.^8^^,^^9^^,^^10^^,^^11^ Glass ionomer cements (GICs) and associated materials are rarely used as definitive restorations in UK primary care.^3^ The clinical studies involving GICs show promise; however, they are primarily short-term and indications are limited.^7^^,^^8^

Though relatively recent economic evaluation data are available from other countries,^12^^,^^13^ studies have taken just one perspective, that of the funder or patient, and do not reflect practising arrangements in England.^8^ The studies have focused on limited outcomes, solely or primarily restoration longevity.^12^^,^^13^ Many other aspects of a restoration are important to patients beyond longevity;^14^ therefore, not considering these characteristics could reduce uptake of services.^8^^,^^15^ This may result in negative downstream effects, such as pain and absenteeism from work, affecting both patients and society at large. Equally, the ability, willingness and capacity of the available clinicians to safely provide an intervention should be considered, but this perspective has been neglected.^8^ All of these broader elements should be considered by policymakers.

Traditional health economic evaluations, including cost-effectiveness, cost-utility and cost-benefit analyses have limitations. They incorporate either select consequences and costs, or combine all of them into a single economic outcome measure. These evaluations can be complex to understand and they may be ignored.^16^

Where broad and varying costs and consequences of healthcare interventions exist from differing perspectives, they can be transparently listed in a cost-consequence analysis as recommended by the National Institute for Health and Care Excellence (NICE).^17^ A decision can then be made which considers all stakeholders and weights the different elements based on the policymaker's preferences. It is also useful for clinical decision-making, allowing clinicians to understand the comprehensive effects of treatments, balancing clinical outcomes with patient preferences and resource use.^13^

Relevant work undertaken to inform this cost-consequence analysis includes: 1) a survey of over 1,500 UK primary care clinicians, looking at material provision and techniques used for placement of direct posterior restorations, incidence of complications, and perceptions of and confidence in using the differing materials in various situations;^3^^,^^8^^,^^18^ 2) a discrete choice experiment which quantified how a representative sample (n = 1,002) of the UK public value different aspects of direct restorations;^14^ and 3) a decision analytic model which followed 10,000 18-year-olds comparing lifetime restoration survival, time until a direct restoration was no longer possible, number of treatment visits, treatment time, and patient, funder and laboratory costs of amalgam versus the alternatives.^19^

This economic evaluation aimed to synthesise these multiple strands of research alongside other new data to broadly evaluate the comparative costs and consequences of using directly placed amalgam, conventional (hybrid) composite, bulk-fill paste composite and bulk-fill flowable composite to restore posterior permanent teeth in adults. It considered multiple relevant stakeholders, the funder, the patient and the clinician, to more holistically inform the phase-out of amalgam under a cost-consequence analysis framework. The study was set in the English NHS for adult patients taking an extended medical sector perspective with societal considerations.

## Methods

The cost-consequence analysis was developed following best practice guidance provided by the UK Government, NICE and the literature as part of a PhD thesis.^2^^,^^16^^,^^17^^,^^20^^,^^21^^,^^22^ A favourable ethical opinion was obtained from Newcastle University Research Ethics Committee (ref 7262/2018).

### Outcomes

The consequences, or outcomes, of interest were selected from a literature review and qualitative work undertaken in preparation for a discrete choice experiment.^8^^,^^14^^,^^22^ The values for each outcome included in the cost-consequence analysis were drawn primarily from data generated specifically for the cost-consequence analysis from the survey, discrete choice experiment and cost-effectiveness analysis model previously discussed, alongside further work presented here.

The survey provided estimates on time taken to place restorations, including the influence of using recommended techniques for composite restorations and post-operative complications.^3^

The model provided non-discounted estimates on clinical outcomes of initial restoration survival, discounted (3.5%)^17^ and non-discounted estimates of time until direct restorations were no longer possible (based on set model parameters of numbers of replacements possible),^19^ lifetime tooth survival (all based on baseline reintervention data following amalgam placement from a large NHS claims dataset^22^^,^^23^^,^^24^^,^^25^^,^^26^ and applied randomised controlled trial data comparing amalgam and composite survival),^27^^,^^28^ treatment time (based on survey data and expert opinion [three NHS practice owners]),^3^ and number of treatment visits over a lifetime (based on expert opinion as before).^19^ Discounting in economic evaluation attempts to account for our lower valuation of benefits and costs occurring in the future than in the present.^17^

Patient preferences for amalgam and composite restorations were quantified by ascribing levels of attributes (waiting time, clinician type, colour, treatment time, post-operative complications and longevity) for the different restorations and then valuing these using marginal willingness to pay data obtained from a discrete choice experiment.^14^ There were insufficient data on the levels of attributes for restorations placed by dental therapists which could feasibly be different,^3^^,^^22^ so it was only performed for treatment by dentists. Estimates on restoration longevity were obtained from a decision analytic model^19^ and treatment times from survey data.^3^ Expert opinion was sought about post-operative complications and waiting time for the differing restorations from the research team and NHS practice owners (n = 8) by email (see online Supplementary Information), and averages and marginal willingness to pay values are presented in the online Supplementary Information (Supplementary Tables 1, 2 and 3).^22^

### Costs

A variety of costing approaches are listed, including the direct costs to the patient and funder obtained from the model based on NHS unit of dental activity 2023 costs,^19^^,^^29^^,^^30^^,^^31^ indirect patient costs, NHS dental practice costs, practice recommended techniques costs and consumable costs.

#### Indirect patient costs

Estimates of treatment time for the compared restorations (and the difference between two bulk-fill materials and conventional composites) over a lifetime were obtained from the model.^19^ These data were used to estimate the indirect costs (patient productivity loss) and marginal (difference in) indirect costs of the various restorations to patients associated with differences in treatment time by multiplying the UK mean pay/minute by the total or marginal treatment time.^29^ In 2023, the UK mean hourly pay for full time workers was £20.83 (£0.35/minute).^32^

#### NHS dental practice costs

Treatment time can also be used to estimate NHS dental practice/dentist costs by using a top-down costing approach.^29^ This uses nationally-determined unit cost data for dental practice time (including, for example, dentist, dental care professional, other staff, consumable and overhead costs)^33^ which are multiplied by the treatment time. The unit cost data are published annually by the Personal Social Services Research Unit providing different estimates for practice owners and associates (£150/hour and £108/hour, respectively).^33^ The number of each of these workers in the NHS dental workforce (2022/23) was 4,604 and 19,512, respectively equating to 19.1% and 80.9%.^31^ Weighting the costs based on this proportion provides an average cost of delivering the dental service of £1.93/minute ([(0.191 × 150) + (0.809 × 108)]/60).

Recommended techniques for placing composite, which were not, however, commonly performed by UK clinicians (especially NHS practitioners), included the use of rubber (dental) dam, sectional matrices, wedges and no liner.^3^^,^^8^^,^^34^^,^^35^ Commonly using these recommended techniques was shown to take more time.^3^ Adding these differences for each technique provides the total extra time required to use the recommended techniques for 2- and 3-surface restorations as shown in Table 1.^22^ It is inappropriate to extrapolate the differences for extra time and costs when using recommended composite techniques, as they would likely result in superior clinical outcomes, and the model used does not change to reflect this.^19^ The clinical data required to re-parameterise the model to reflect this difference is not available.

**Table 1:** Extra time taken for composite restorations using recommended techniques frequently versus not^22^

**Recommended technique** **(high use)**	**Extra time taken (minutes) (95% confidence interval)**	
	**2-surface composite (n = 777)**	**3-surface composite (n = 769)**
Rubber dam	5 (2 − 9)	8 (4 − 12)
Sectional matrix	3 (0 − 6)	4 (0 − 8)
Wedge	2 (0 − 3)	2 (0 − 3)
No liner	1 (-1 − 2)	1 (-1 − 3)
All (total)	10 (1 − 20)	15 (3 − 26)
Results presented to the nearest minute		

#### Consumable costs

The costs of consumables to the practice or clinician per restoration were taken from the Henry Schein (dental equipment and material supplier) website (www.henryschein.co.uk) accessed August 2023. Where available, at least three alternatives for each consumable product used in each restoration were listed alongside their prices. Mean average costs were calculated (aside from when costing cheapest or own brand alternatives). Maximum and minimum costs of each consumable were also recorded (except for cheapest or own brand alternatives). The minimum, maximum and mean consumable material costs for each restoration were then calculated and VAT added (online Supplementary Tables 4–10 show itemised costs/restoration),^22^ which included amalgam disposal costs. Recommended composite restoration costs included the use of sectional matrices, rubber dam and no liner, and branded restorative materials only, whereas an average conventional composite involved the use of a Tofflemire matrix band, cotton wool rolls, a saliva ejector and a setting calcium hydroxide liner, (based on the most common use of materials and equipment by UK clinicians when performing composite restorations)^3^^,^^19^^,^^22^ and both branded and unbranded restorative materials. Table 2 provides a summary of these costs/restoration.^22^

**Table 2:** Consumable costs for each restoration type^22^

**Material**	**Cost/restoration (with 20% VAT) (£)**		
	**Mean**	**Minimum**	**Maximum**
Amalgam	5	3	6
Basic (own brand) conventional composite	7^†^	-	-
Average conventional composite	12	7	17
Recommended conventional composite	21	17	27
Basic (own brand) bulk-fill flowable composite	11^†^	-	-
Recommended bulk-fill flowable composite	22	18	27
Recommended bulk-fill paste composite	16	12	21
^†^ = lowest cost. VAT, value added tax. Results presented to the nearest pound			

#### Non-consumable costs

It was assumed that non-consumable costs of providing each restoration were similar with negligible difference for each material. Extended patient/provider safety costs and cremation and environmental costs were not newly estimated for this cost-consequence analysis but estimates from the work of others have been provided elsewhere and these were used to inform the cost-consequence analysis.^22^

## Results

Table 3 and Table 4 summarise the costs and consequences of using amalgam restorations and alternatives.^22^ A summary of the differences between composite restoration materials and techniques is provided in Table 5.^22^ All values were rounded to the nearest integer, with unrounded values used in calculations. 95% confidence intervals and prediction intervals showed very little variation for all modelled parameters (other than marginal willingness to pay) so they are not presented in the tables.

**Table 3:** Outcome differences between amalgam and composite direct posterior restorations^22^

**Outcomes**				**Amalgam**	**Composite**	**Difference†**
Clinical		Restoration survival^D^ (years)		11	7	-4
		Restoration survival^ND^ (years)		17	9	-9
		Tooth survival^D^ (years)		21	17	-4
		Tooth survival^ND^ (years)		44	32	-12
		Time until direct restorations were no longer possible^D^ (years)		18	12	-6
		Time until direct restorations were no longer possible^ND^ (years)		35	18	-17
		Post-operative complications (%)^‡^	None	76	58	Greater risk composite. Higher for NHS dentists and therapists
			Mild	16	25	
			Moderate	6	12	
			Persistent	3	6	
Treatment time (minutes)		2-surface restoration		24	34	10
		Lifetime^D^		64	107	43
		Lifetime^ND^		110	156	46
Treatment visits		2-surface restoration		1	1	0
		Lifetime^D^		3	4	1
		Lifetime^ND^		4	5	1
Patient/public valuation (mWTP) (£) (95% CIs)	General population	Waiting time^‡^		3 (-8 − 15)	-14 (-26 − -3)	-18 (-18 − -17)
		Clinician type		7 (4 – 9)	7 (4 – 9)	0 (0 – 0)
		Colour		-21 (-25 − -17)	21 (17 – 25)	42 (42 – 42)
		Treatment time		-7 (-10 − -4)	-9 (-14 − -5)	-3 (-4 − -1.5)
		Post-operative complications^‡^		39 (32 − 46)	31 (24 − 38)	-8 (-8 − -8)
		Lifespan		93 (77 – 109)	47 (39 – 55)	-47 (-38 − -55)
		Total		115 (71 – 159)	82 (44 – 120)	-33 (-27 − -40)
	Low income	Waiting time^‡^		3 (-14 − 19)	-14 (-31 − 3)	-17 (-17 − -17)
		Clinician type		5 (1 – 9)	5 (1 – 9)	0 (0 – 0)
		Colour		-9 (-15 − -3)	9 (3 – 15)	18 (18 – 18)
		Treatment time		-8 (-13 − -2)	-11 (-18 − -4)	-3 (-5.3 − -1.2)
		Post-operative complications^‡^		29 (17 − 42)	24 (12 – 36)	-6 (-5 − -6)
		Lifespan		53 (29 – 78)	27 (14 – 39)	-27 (-14 − -39)
		Total		74 (5 – 142)	40 (-19 – 97)	-34 (-24 − -45)
	Higher income	Waiting time^‡^		3 (-12 − 18)	-15 (-30 – 0)	-18 (-19 − -17)
		Clinician type		7 (4 – 11)	7 (4 – 11)	0 (0 – 0)
		Colour		-25 (-30 − -20)	25 (20 – 30)	50 (50 – 50)
		Treatment time		-6 (-10 − -2)	-8 (-14 − -3)	-2 (-4 − -1)
		Post-operative complications^‡^		41 (32 – 51)	33 (24 – 42)	-8 (-8 − -8)
		Lifespan		109 (87 – 131)	54 (43 – 66)	-54 (-43 − -65)
		Total		129 (71 – 188)	97 (47 – 146)	-33 (-24 − -42)
^†^ = composite – amalgam using unrounded values; ^‡^ = estimates derived from experts; ^D^ = 3.5% discounted; ^ND^ = no discounting. CIs, confidence intervals; mWTP, marginal willingness to pay

**Table 4:** Cost differences between amalgam and composite direct posterior restorations^22^

**Costs (£)**			**Amalgam**	**Composite**	**Difference**
2-surface restoration	Funder (NHS)	Average patient^†^	24	24	0
		Paying patient	14	14	0
		Exempt patient	59	59	0
	Patient	Direct^‡^	45	45	0
		Indirect	8	12	4
	Dental practice	Overall	46	66	20
		Consumables	5^¶^	12^§^	7
Over lifetime (3.5% discounting)	Funder (NHS)	Average patient^†^	81	115	34
		Paying patient	43	60	17
		Exempt patient	201	290	89
	Patient	Direct^‡^	158	228	70
		Indirect	22	37	15
	Dental practice	Overall	124	207	83
		Laboratory	11	18	8
Environmental impact^#^			Low	Uncertain	Uncertain
Patient/provider safety^#^			Low	Low, uncertain	Minor, uncertain
^†^ = Based on exempt 23.6%: payer 76.4% adult courses of treatment^32^ (example calculation for average NHS cost band 2a = [17.26 × 0.764] + [87.96 × 0.236] = £20.76); ^‡^ = Paying patients only; ^§^ = Minimum composite cost = £7, large increase in costs when using branded composite materials; ^¶^ = Minimum amalgam cost = £3, includes waste disposal fees; ^#^ = Not investigated as original research;^22^ NHS, National Health Service

**Table 5:** Composite restoration time and cost differences with recommended techniques or bulk-fill materials^22^

**Composite technique/material**	**Extra time (minutes) (95% CIs)**		**Extra cost (£) (95% CIs)**				
			**Patient indirect**		**Dentist** **consumables**	**Dental practice overallβ**	
	**Per 2-surface restoration**	**LifetimeD**	**Per 2-surface restoration**	**LifetimeD**	**Per 2-surface restoration**	**Per 2-surface restoration**	**LifetimeD**
Recommended techniques versus not	10 (1 – 20)	^†^	3	^†^	10	20 (2 – 39)	^†^
Conventional versus bulk-fill flowable	2	3	1	1	0^‡^ -4^§^	3	6
Conventional versus bulk-fill paste	2	4	1	1	6^‡^	5	8
^D =^ 3.5% discounted; ^† =^ inappropriate to extrapolate additional time and costs over a lifetime when using recommended techniques with the current model given the likely improved outcomes; ^‡ =^ recommended technique and branded material restorations; ^§ =^ basic (own brand) materials. CIs, confidence intervals							

All outcomes, in terms of restoration and tooth survival, post-operative complications, treatment time, visits and patient or public valuations, favour amalgam. The general population, and when split into low- and higher-income groups, valued amalgam more than composite restorations by £33 or £34. However, the low-income group valued amalgam 1.9 times more than composite restorations, and this was higher than the general population (1.4) and higher-income group (1.3). Public valuation differences between conventional and bulk-fill composites were negligible.

All costs, for patient (including direct and indirect costs), dental practice/clinician and funder were higher for composite (other than funder costs for initial restoration). Using recommended techniques and branded materials for composite restorations was much more time consuming and costly for dental practices/clinicians.

## Discussion

### Outcomes and costs

This cost-consequence analysis, which compares direct restorations of composite and amalgam, draws on previous work, including a survey, discrete choice experiment and cost-effectiveness analysis model, and expands it to summarise and quantify the differences for multiple consequences and stakeholders. Apart from restoration appearance, where the public valued composite more than amalgam, all other costs and outcomes quantified favour amalgam use for patients, clinicians and funders. Time until a direct restoration is no longer possible is important, as many people do not have indirect restorations placed on root-filled^27^ or heavily compromised teeth possibly because of the expense involved.^18^ This difference between materials may therefore primarily impact those of low socio-economic status who could not afford more expensive treatment, potentially resulting in more teeth being extracted in this group, exacerbating existing health inequalities.^8^^,^^18^ Lifetime average costs were much higher for composite than amalgam for all stakeholders, but cost differences were higher for the patient in comparison to the funder, and higher still for the dental practice or clinician.

Given that in the English NHS, remuneration is the same for all direct restorations and is much lower than EU or private remuneration,^8^ but composite incurs higher time, consumable, clinician and practice costs, the health system essentially disincentivises its use as supported by material usage data.^3^^,^^8^ This in turn then also disincentivises clinicians to upskill to use recommended, but technically demanding, techniques for composite restorations^8^ as they incur much higher time (£20) and consumable (£10) costs for a two-surface restoration than when not using these techniques. These recommended techniques do however reduce reported post-operative complications^3^ which is highly valued by the public.^14^ It would be interesting to perform a similar study modelling the use of recommended techniques for composite placement but there would be much uncertainty.

The findings in this study are broadly the same as the only other cost-consequence analysis comparing amalgam and composite restorations, albeit in the Canadian setting.^13^ The presented study includes broader consequences using a more sophisticated model to estimate lifetime outcomes for more consequences and costs to more stakeholders; although, the Canadian study did estimate the mercury waste management costs which this study did not.^13^^,^^36^

### Extended differences

A number of differences between the materials are not accounted for in this cost-consequence analysis, which tend to underestimate the dominance of amalgam over composite and include:

- Consumable costs over a lifetime given the increased unit cost and need for replacement with composite, alongside the increased amount of relatively more complex, more expensive treatment required. The use of untested own brand composite materials and bonding agents in NHS primary care is uncertain, but likely significant given the potential short-term cost savings. Their efficacy and use is an area for further investigation
- Indirect and direct patient costs of travelling to and from appointments were not calculated but would be higher for composite given the increased numbers of visits. This would likely also impact on carbon emissions and therefore the environment and sustainability, which would be beneficial to quantify
- Emergency costs would likely be higher for composite due to the increased number of interventions and self-medication (analgesics for post-operative pain for example)
- Removal of a failed composite compared to a failed amalgam restoration generally takes more time and the sound tooth tissue removed is increased,^37^ again increasing costs and potentially further negatively impacting survival for composites
- Clinician confidence is much lower when placing composite in difficult situations (e.g., managing cavities with deep subgingival margins) than it is with amalgam.^4^^,^^18^ This is despite a large majority having received postgraduate training on posterior composites, and the existence of techniques to predictably manage these situations.^38^^,^^39^^,^^40^^,^^41^ This suggests a failure of postgraduate education which needs to be addressed
- The cost-consequence analysis therefore does not fully capture the likely increased need for extraction of teeth where amalgam is not available. Some teeth will be deemed unrestorable without amalgam as it is much more tolerant of contamination during the procedure or more expensive indirect restorations.^8^^,^^18^ This may also lead to earlier intervention with them
- Phasing-in alternatives inevitably results in up-front costs, which relate to administration and implementation, alongside dental education costs which have not been considered^12^
- These differences would likely disproportionately affect those with higher need in society who cannot afford more expensive interventions.

However, the costs of amalgam are likely to rise as manufacture becomes more limited and transport costs to England rise. Equally with more regular use of composite, treatment time may reduce and outcomes may improve.

### Broader context

A total of 4.3 million Band 2 courses of treatment with ‘permanent' fillings were recorded in adults in England (2022–23), from 5.1 million total courses of treatment with ‘permanent' fillings included.^31^ This does not detail the number of restorations delivered, or whether the restorations were posterior, anterior or both, making application of these findings to existing English NHS dental services difficult. Central data collection could be hugely improved to facilitate planning of future dental services.

The data suggest that an amalgam phase-out would inevitably lead to a reduced capacity to treat the same levels of disease with the same workforce, even without the expectation of more clinicians leaving the NHS.^42^

To retain an NHS service, the clinician perspective must be considered. This would likely require significant increases in remuneration and changes in structuring of the NHS dental services (including a focus on prevention). Without fundamental changes a crisis looms, which would primarily affect the most at need in society, exacerbating existing health inequalities.

### Limitations

The datasets used to provide the estimates in the cost-consequence analysis have limitations as previously described.^3^^,^^14^^,^^18^^,^^19^^,^^22^ More detailed costings could be provided by using a micro-costing approach. Consumable costs were only calculated for the initial restoration, so extending these to all modelled interventions would provide an estimate of the total consumable costs over a lifetime. Specific non-consumable costs were not estimated. Expert opinion was used to parameterise two attributes' levels of the different restorations for the public valuation. Data on the environmental impact of composites are very limited and therefore the difference in costs is unknown.^8^^,^^13^^,^^36^^,^^43^ Further work to elucidate these would be helpful.

## Conclusions

This cost-consequence analysis, taking an extended English NHS medical sector perspective with societal considerations, showed that, aside from appearance, all other costs and outcomes favoured amalgam use over composites when directly restoring posterior teeth for all stakeholders. If amalgam is phased out in the near future without fundamental change, there are significant implications for the most at need in society and the survival of NHS dental services.

## Supplementary Information

Supplementary Information (DOCX 34KB)

## Data Availability

Data will be made available upon reasonable request to the corresponding author.
