# Supplementary material for: Amalgam versus composite restorations: a cost-consequence analysis
Source: Br Dent J. 2026 Jul 24;241(2):128–34. doi: 10.1038/s41415-026-9797-1 (PMC13400300; doi:10.1038/s41415-026-9797-1)
Supplement: Supplementary file 1 — Supplementary Information (DOCX 34KB) [file 41415_2026_9797_MOESM1_ESM.docx]

**Amalgam vs composite restorations: a cost-consequence analysis**

Bailey O, Ternent L, Stone S, Vernazza C

**Appendix**

(all material included from PhD thesis: Bailey O. Amalgam phase-out: what next for dentistry? The costs and benefits of the alternative direct restorations [dissertation]. Newcastle upon Tyne (UK): Newcastle University; 2025.)

Expert opinion request email:

Dear all,

I'd appreciate it if you could take a couple of minutes and fill in the following information (highlighted) which would be really helpful for my PhD. I'm trying to get a rough idea of expert opinion. The values are not specific to you, but what you feel the values would generally be for NHS primary care dentists restoring posterior teeth in adult patients. I'll also be pooling the answers and not recording your name so they'll be anonymised. Could you reply just to me please, so you don't influence others. I'd be grateful if you could respond by 6^th^ September.

Likely waiting time for a restoration (within an NHS practice setting) (0,2,4 or 6 weeks)

Composite =

Amalgam =

And

Post operative complications (in NHS primary dental care)

**The levels were: none, mild, moderate, persistent.**

This relates to the likely level of discomfort when eating and drinking after having a filling placed.

None - self-explanatory
Mild (short-lived low-level sensitivity for 2-4 weeks not causing problems with function)
Moderate (requiring painkillers and may mean that you would avoid eating, chewing or drinking certain foods or drinks for 2-4 weeks)Persistent (requiring reattendance at the dental practice for the management of a problem after 2-4 weeks)

Could you record a percentage of each option for each restoration type adding up to 100% for each material please

Composite

None =

Mild =

Moderate =

Persistent =

Amalgam

None =

Mild =

Moderate =

Persistent = 

**Appendix Table 1. Expert opinion on post-operative complication incidence for restoration materials in NHS primary care relating to discrete choice experiment levels.**

| **Expert** | **Post-operative complications (%)** | | | | | | | |
| --- | --- | --- | --- | --- | --- | --- | --- | --- |
|  | **Amalgam** | | | | **Composite** | | | |
|  | **None** | **Mild** | **Moderate** | **Persistent** | **None** | **Mild** | **Moderate** | **Persistent** |
| 1 | 70 | 20 | 5 | 5 | 50 | 25 | 15 | 10 |
| 2 | 80 | 10 | 7 | 3 | 50 | 30 | 20 | 10 |
| 3 | 95 | 2 | 1 | 1 | 90 | 6 | 2 | 2 |
| 4 | 95 | 3 | 2 | 1 | 80 | 10 | 8 | 2 |
| 5 | 90 | 7 | 2 | 1 | 70 | 15 | 10 | 5 |
| 6 | 75 | 15 | 7 | 3 | 60 | 25 | 10 | 5 |
| 7 | 40 | 40 | 15 | 5 | 25 | 50 | 20 | 5 |
| 8 | 60 | 30 | 5 | 5 | 40 | 40 | 10 | 10 |
| Average | 76 | 16 | 6 | 3 | 58 | 25 | 12 | 6 |

Averages given to nearest integer.

**Appendix Table 2. Post-operative complication marginal willingness to pay for different restoration materials in NHS primary care relating to discrete choice experiment levels based on expert opinion**

| **Post-operative complications** | **Amalgam** | | | | **Composite** | | | |
| --- | --- | --- | --- | --- | --- | --- | --- | --- |
|  | **Proportional incidence**^†^ | **mWTP * proportion (£) (95% CIs)** | | | **Proportional incidence**^†^ | **mWTP * proportion (£) (95% CIs)** | | |
|  |  | **General population** | **Low-income** | **Higher-income** |  | **General population** | **Low-income** | **Higher-income** |
| None | 0.76 | 35 (30 - 41) | 26 (17 - 36) | 37 (30 - 44) | 0.58 | 27 (23 - 31) | 20 (13 - 28) | 28 (23 - 34) |
| Mild | 0.16 | 7 (6 - 8) | 5 (4 - 7) | 8 (6 - 9) | 0.25 | 11 (9 - 13) | 9 (6 - 11) | 12 (10 - 14) |
| Moderate | 0.06 | -1 (-2 - -1) | -1 (-1 - 0) | -1 (-2 - -1) | 0.12 | -2 (-3 - -2) | -2 (-3 - -1) | -3 (-3 - -2) |
| Persistent | 0.03 | -2 (-2 - -2) | -2 (-2 - -1) | -2 (-3 - -2) | 0.06 | -4 (-5 - -4) | -3 (-4 - -2) | -4 (-5 - -4) |
| Average | N/A | 39 (32 - 46) | 29 (17 - 42) | 41 (32 - 51) | N/A | 31 (24 - 38) | 24 (12 - 36) | 33 (24 - 42) |

mWTP * proportion, marginal willingness to pay of value for each complication level multiplied by the proportional incidence for each restoration; ^†^unrounded values used to calculate mWTP; 95% CIs, 95% confidence intervals; mWTP values given to nearest integer.

Appendix Table 3. Expert opinion on waiting times converted to marginal willingness to pay values for different restoration materials in NHS primary care relating to discrete choice experiment levels

| **Expert** | **Waiting time (weeks)** | | | | | | | |
| --- | --- | --- | --- | --- | --- | --- | --- | --- |
|  | **Amalgam** | | | | **Composite** | | | |
|  | **Weeks** | **mWTP (£) (95% CIs)** | | | **Weeks** | **mWTP (£) (95% CIs)** | | |
|  |  | **General population** | **Low-income** | **Higher-income** |  | **General population** | **Low-income** | **Higher-income** |
| 1 | 4 | 3 (-8 - 14) | -4 (-20 - 13) | 4 (-10 - 18) | 4 | 3 (-8 - 14) | -4 (-20 - 13) | 4 (-10 - 18) |
| 2 | 2 | 19 (9 - 30) | 23 (6 - 39) | 18 (4 - 32) | 6 | -20 (-32 - -8) | -17 (-35 - -1) | -21 (-37 - -6) |
| 3 | 6 | -20 (-32 - -8) | -17 (-35 - -1) | -21 (-37 - -6) | 6 | -20 (-32 - -8) | -17 (-35 - -1) | -21 (-37 - -6) |
| 4 | 4 | 3 (-8 - 14) | -4 (-20 - 13) | 4 (-10 - 18) | 4 | 3 (-8 - 14) | -4 (-20 - 13) | 4 (-10 - 18) |
| 5 | 4 | 3 (-8 - 14) | -4 (-20 - 13) | 4 (-10 - 18) | 6 | -20 (-32 - -8) | -17 (-35 - -1) | -21 (-37 - -6) |
| 6 | 2 | 19 (9 - 30) | 23 (6 - 39) | 18 (4 - 32) | 6 | -20 (-32 - -8) | -17 (-35 - -1) | -21 (-37 - -6) |
| 7 | 6 | -20 (-32 - -8) | -17 (-35 - -1) | -21 (-37 - -6) | 6 | -20 (-32 - -8) | -17 (-35 - -1) | -21 (-37 - -6) |
| 8 | 2 | 19 (9 - 30) | 23 (6 - 39) | 18 (4 - 32) | 6 | -20 (-32 - -8) | -17 (-35 - -1) | -21 (-37 - -6) |
| Average | 4 | 3 (-8 - 15) | 3 (-14 - 19) | 3 (-12 - 18) | 6 | -14 (-26 - -3) | -14 (-31 - 3) | -15 (-30 - 0) |

95% CIs, 95% confidence intervals; mWTP, marginal willingness to pay; unrounded mWTP values used; results given to nearest integer.

Appendix Table 4. Generic restoration consumable costs (same for all restorations)

| **Material (generic)** | **Cost/restoration (with 20% VAT) (£)** | | |
| --- | --- | --- | --- |
|  | **Mean** | **Minimum** | **Maximum** |
| Local anaesthetic solution | 0.60 | 0.56 | 0.62 |
| Local anaesthetic disposable barrel | 0.36 | 0.36 | 0.36 |
| Bib | 0.12 | 0.06 | 0.19 |
| Disposable cup | 0.04 | 0.02 | 0.05 |
| Tray cover | 0.08 | 0.04 | 0.15 |
| 3-in-1 tip | 0.29 | 0.08 | 0.48 |
| Suction tip | 0.06 | 0.05 | 0.09 |
| Mask IIR (operator and assistant) | 0.14 | 0.12 | 0.19 |
| Gloves nitrile (operator and assistant | 0.23 | 0.16 | 0.29 |
| Articulating paper | 0.07 | 0.04 | 0.11 |
| Total (unrounded values) | 1.99 | 1.49 | 2.54 |

Appendix Table 5. Amalgam consumable costs

| **Material** | **Cost/restoration (with 20% VAT) (£)** | | |
| --- | --- | --- | --- |
|  | **Mean** | **Minimum** | **Maximum** |
| Amalgam (2 spill) | 1.76 | 1.37 | 1.99 |
| Calcium hydroxide lining | 0.05 | 0.04 | 0.07 |
| Tofflemire matrix band | 0.48 | 0.18 | 0.91 |
| Wooden wedge | 0.21 | 0.14 | 0.25 |
| Cotton wool rolls | 0.06 | 0.03 | 0.09 |
| Liner | 0.05 | 0.03 | 0.07 |
| Amalgam capsule waste storage^†^ | 0.15 | 0.13 | 0.17 |
| Generic disposables | 1.99 | 1.49 | 2.54 |
| Total (unrounded values) | 4.74 | 3.40 | 6.08 |

^†^Based on 4-5 restorations performed/day NHS practice, 5 working days and 47 working weeks per year (N. Diddee (Clinical director Riverdale corporate group), private communication, August 2024) = 1057.5 (range 940-1175) amalgam restorations/year.

Amalgam waste pots cost: £131.04 per surgery per year (2 of each 500ml and Bulk pot per surgery per year (includes disposal cost) (N Diddee, personal communication, May 2024). With 20% VAT = £157.25.

Therefore disposal cost/amalgam restoration = £157.25/1057.5 = £0.15

Minimum: £157.25/1175 = £0.13

Maximum: £157.25/940 = £0.17

Appendix Table 6. Average conventional composite consumable costs

| **Material** | **Minimum cost/restoration (with 20% VAT) (£)** | | |
| --- | --- | --- | --- |
|  | **Mean** | **Minimum** | **Maximum** |
| Conventional paste composite | 5.11 | 2.19 | 6.78 |
| Calcium hydroxide lining | 0.05 | 0.04 | 0.07 |
| Phosphoric acid etch gel + tips | 0.62 | 0.62 | 0.62 |
| Bonding agent | 2.50 | 0.98 | 3.58 |
| Microbrushes | 0.54 | 0.22 | 0.82 |
| Finishing discs | 0.65 | 0.49 | 0.8 |
| Light curing shield | 0.08 | 0.08 | 0.08 |
| Tofflemire matrix | 0.48 | 0.18 | 0.91 |
| Wooden wedge | 0.21 | 0.14 | 0.25 |
| Cotton wool rolls | 0.06 | 0.03 | 0.09 |
| Saliva ejector | 0.05 | 0.05 | 0.05 |
| Generic disposables | 1.99 | 1.49 | 2.54 |
| Total | 12.34 | 6.50 | 16.59 |

**Appendix Table 7. Recommended conventional composite consumable costs**

| **Material (conventional composite ‘recommended’ with branded material)** | **Cost/restoration (with 20% VAT) (£)** | | |
| --- | --- | --- | --- |
|  | **Mean** | **Minimum** | **Maximum** |
| Conventional paste composite | 6.09 | 5.22 | 6.78 |
| Conventional flowable composite | 4.94 | 3.81 | 6.26 |
| Phosphoric acid etch gel + tips | 0.62 | 0.62 | 0.62 |
| Bonding agent | 2.89 | 2.47 | 3.58 |
| Microbrushes | 0.54 | 0.22 | 0.82 |
| Finishing discs | 0.65 | 0.49 | 0.80 |
| Light curing shield | 0.08 | 0.08 | 0.08 |
| Sectional matrix | 1.80 | 1.04 | 3.46 |
| Plastic wedge | 0.56 | 0.50 | 0.62 |
| Rubber/dental dam (latex free) | 1.29 | 1.08 | 1.74 |
| Generic disposables | 1.99 | 1.49 | 2.54 |
| Total | 21.43 | 17.02 | 27.30 |

**Appendix Table 8. Recommended bulk-fill paste composite consumable costs**

| **Material** | **Cost/restoration (with 20% VAT) (£)** | | |
| --- | --- | --- | --- |
|  | **Mean** | **Minimum** | **Maximum** |
| Bulk-fill paste composite | 5.51 | 3.78 | 6.40 |
| Phosphoric acid etch gel + tips | 0.62 | 0.62 | 0.62 |
| Bonding agent | 2.89 | 2.47 | 3.58 |
| Microbrushes | 0.54 | 0.22 | 0.82 |
| Finishing discs | 0.65 | 0.49 | 0.80 |
| Light curing shield | 0.08 | 0.08 | 0.08 |
| Sectional matrix | 1.80 | 1.04 | 3.46 |
| Plastic wedge | 0.56 | 0.50 | 0.62 |
| Rubber/dental dam (latex free) | 1.29 | 1.08 | 1.74 |
| Generic disposables | 1.99 | 1.49 | 2.54 |
| Total | 15.92 | 11.77 | 20.66 |

Appendix Table 9. Recommended bulk-fill flowable composite consumable costs

| **Material** | **Cost/restoration (with 20% VAT) (£)** | | |
| --- | --- | --- | --- |
|  | **Mean** | **Minimum** | **Maximum** |
| Bulk-fill flowable composite | 5.35 | 4.89 | 6.00 |
| Conventional paste composite | 6.09 | 5.22 | 6.78 |
| Phosphoric acid etch gel + tips | 0.62 | 0.62 | 0.62 |
| Bonding agent | 2.89 | 2.47 | 3.58 |
| Microbrushes | 0.54 | 0.22 | 0.82 |
| Finishing discs | 0.65 | 0.49 | 0.80 |
| Light curing shield | 0.08 | 0.08 | 0.08 |
| Sectional matrix | 1.80 | 1.04 | 3.46 |
| Plastic wedge | 0.56 | 0.50 | 0.62 |
| Rubber/dental dam (latex free) | 1.29 | 1.08 | 1.74 |
| Generic disposables | 1.99 | 1.49 | 2.54 |
| Total | 21.84 | 18.10 | 27.04 |

Appendix Table 10. Basic (own brand) bulk-fill flowable composite consumable costs

| **Material (bulk-fill flowable composite basic)** | **Minimum cost/restoration (with 20% VAT) (£)** |
| --- | --- |
| Bulk-fill flowable composite | 4.16 |
| Conventional paste composite | 2.19 |
| Phosphoric acid etch gel + tips | 0.62 |
| Bonding agent | 0.98 |
| Microbrushes | 0.22 |
| Finishing discs | 0.49 |
| Light curing shield | 0.08 |
| Tofflemire matrix | 0.18 |
| Wooden wedge | 0.14 |
| Cotton wool rolls | 0.03 |
| Saliva ejector | 0.05 |
| Generic disposables | 1.49 |
| Total | 10.62 |
